# Artificial Intelligence in Exams by Image: Ethical Pros and Cons

**DOI:** 10.3390/healthcare14091240

**Published:** 2026-05-04

**Authors:** Caio Zachini, Sofia Nunes, Francisca Rego, Rui Nunes

**Affiliations:** RISE-Health, Faculty of Medicine, University of Porto, Alameda Prof. Hernâni Monteiro, 4200-319 Porto, Portugal; asnunes@med.up.pt (S.N.); mfrego@med.up.pt (F.R.); ruinunes@med.up.pt (R.N.)

**Keywords:** artificial intelligence, bioethics, computer-assisted image analysis, medicine, radiological reports

## Abstract

**Background/Objectives**: The world’s interest in the applications of artificial intelligence (AI) is rapidly increasing. In medicine, machine learning-based devices have proliferated, especially for image analysis, heralding significant new challenges for the use of AI in healthcare. Based on this context, this research addresses the following question: What are the major bioethical issues related to the use of AI in radiological patient reports? **Methods**: This study examined the main bioethical concerns surrounding AI in radiological reports, based on a narrative literature review grounded in the works of prominent authors in the field of bioethics. **Results**: We highlight the legal frameworks regulating medical devices and data protection in the European Union and the United States of America, evaluating recent developments in the contemporary medical landscape. **Conclusions**: Despite progress, many issues remain unresolved and must be addressed in order to advance the regulation of AI applications in medicine.

## 1. Introduction

Every human being is susceptible to failures, and one of the fields most committed to minimizing the occurrence of errors is medicine. Even with extensive knowledge regarding pathologies and by considering large amounts of information about symptoms, causes and patients’ medical histories, professionals involved in medical procedures can make mistakes during evaluations, which may influence the decision-making process. These issues can negatively impact intervention in serious pathologies, for example, and in certain situations, proper therapy is not administered in time to prevent further development of the disease.

With the development of medicine, an unprecedented proliferation of scientific knowledge has occurred, but not all accumulated knowledge is always assimilated by the medical community. At the forefront of this issue, contemporary society witnesses the increasing importance of extremely rapid and precise diagnoses as a response to the ever-growing demands of the health sector. Nowadays, the phenomenon of mass dissemination and sharing of health-related information is evident; however, an algorithm capable of efficiently scanning these as a network of symptoms to generate a concise diagnosis for an individual patient has not been developed. It is pertinent to mention that part of this challenge is linked to the unique characteristics of each individual; however, a timely diagnosis can mean the difference between life and death [1].

With a preventive approach, artificial intelligence (AI) has been strategically applied in the medical field to improve a series of processes and results, with an emphasis on enhancing efficiency, agility and overall quality. It is believed that AI, when provided with a database of medical information, can contribute substantially to treatments [2]. Numerous studies aim to apply AI in medicine by utilizing support systems to aid medical decision-making. However, these systems are still not being implemented as widely as expected. There are many questions related to this issue: the prior need for computerized medical history systems; complex user interfaces; excessive time spent in initial patient registration; and, in many situations, the need for and immediate response from the medical professional [3].

AI had its breakthrough approximately 50 years ago, and its initial goal was to develop computerized structures that imitate the information processing of human beings [2]. It is a branch of computer science focused on creating systems that perform tasks typically requiring human intelligence, using various technical approaches [4]. The term “artificial intelligence” refers to computer systems that imitate cognitive functions, such as learning and problem solving [5]. These technologies are present in many areas of knowledge, involving a variety of specific techniques and procedures, such as fuzzy logic, Bayesian networks and artificial neural networks. Such systems rely on flexible mathematical models and employ various algorithms to identify complex, nonlinear relationships within large datasets, commonly referred to as Big Data [6].

Big Data encompasses information from mobile applications, wearable technology, social media, environmental and lifestyle factors, social and demographic aspects, economic data, and electronic health records, among others [7]. Within the machine learning domain, deep learning stands out as a highly promising technique, particularly for image processing [8]. Unlike software learning, which requires specific instructions to complete a task, deep learning enables systems to autonomously identify patterns and make effective predictions [9]. The application of deep learning to Big Data has exerted a significant influence on medical decision-making [10,11]. However, challenges related to the adoption of AI in medicine, and specifically in radiology, are less dependent on physicians and more influenced by regulatory institutions and government authorities in each country [12].

Even though it remains an area requiring further research and development, AI applied in medicine shows significant potential to enhance how doctors evaluate situations and make decisions. Since its earliest iterations, this technology has delivered substantial results and heightened expectations regarding its application in the medical field [13]. In the context of medical imaging, artificial intelligence is defined as a branch of computer science capable of creating algorithms that, through machine learning and deep learning, identify complex patterns within radiological datasets to assist in tasks such as lesion detection, tissue characterization, and automated measurement. This technology functions not merely as a tool for image enhancement, but as a decision-support system that emulates human cognitive functions in interpreting high-dimensional data [14].

Based on this context, the present research highlighted the following question: What are the main bioethical issues related to the use of AI in radiological patient reports? Regarding the general objective, this study analyses the key characteristics of AI in medicine, with particular focus on the field of radiology. The specific objectives include: describing the features related to the application of AI in medicine; characterizing the regulatory aspects governing its use in the European Union and in the United States of America; and analyzing the bioethical challenges arising from the deployment of AI in medicine in these regions.

## 2. Materials and Methods

This study was based on exploratory qualitative research conducted through a narrative literature review. This approach was chosen to allow for a critical and discursive synthesis of the complex intersection between bioethics, law, and technology, favoring theoretical depth and the integration of different fields of knowledge. The nature of qualitative research presents the following characteristics: it has the natural environment as a direct source of data, with the researcher acting as the main instrument; the collected data is predominantly descriptive; there is a greater concern with the process rather than the product; the meaning that people give to things and to their lives is the focus of special attention by the researcher; and the data analysis tends to follow an inductive process [15,16].

The selection of sources followed a non-systematic, purposive logic, focusing on documents and authors with recognized institutional authority and relevance to the research problem. The search included primary legal sources, such as the EU AI Act and FDA regulations, alongside seminal works in bioethics and clinical studies in radiology. The theoretical foundation of this research was established through consultations of books, academic journals, scientific articles, and reputable scientific websites. The bibliographic review provided essential theoretical support for the analysis of relevant issues and the precise definition of key terms related to the research area. It is a critical, meticulous and comprehensive analysis of current publications in a given area of knowledge. In this sense, the careful selection of the pertinent literature for the problem means familiarizing oneself with texts and, through them, recognizing the authors and their previous studies about the researched problem [17].

The analytical process was inductive and interpretative, focused on identifying and categorizing recurring themes such as algorithmic opacity, data protection, and professional liability. The results obtained from the exploratory qualitative research were categorized and are presented below: in the next section, characteristics related to the use of AI in medicine are described; in the subsequent section, bioethical questions and regulations regarding the use of AI in medicine are analyzed in European countries and the United States; and in a later section, the final considerations are presented, where the conclusions of this study are provided.

## 3. The Use of Artificial Intelligence in Medicine

Medicine is the field responsible for preserving and promoting health, always aiming at improving wellbeing. The current conceptualization of medicine refers to a set of practices, approaches, and knowledge that integrates both material and mental concepts, practical techniques and exercises. These methods may be used individually or in combination, either for individuals or larger communities, with the purpose of diagnosing, treating, and preventing diseases. The doctor’s function is to investigate and study diseases and their causative agents to better combat, cure and/or develop effective prevention strategies. To achieve this, the professional must stay constantly updated on new technologies and information resources that can enhance their work, ensuring higher standards of quality and effectiveness [18].

Medicine is a highly complex field that has evolved significantly, particularly in terms of technique, and its methods have been expanding at an increasingly rapid pace. To a large extent, the transformations witnessed in medicine are closely related to the advancement of new technologies. From this perspective, interdisciplinary collaboration between medical professionals and computer scientists is becoming ever more essential, driven by intelligent technologies that provide new tools for the optimization of medical processes [19].

Scientific and technological developments are progressing at an accelerated and continuous pace. Among the various technological fields, AI has gained considerable emphasis as it aims to support health professionals in their activities, assisting with tasks that require the analysis of complex data and the application of specialized knowledge. Initially, scientists focused on developing AI for supporting diagnosis and making therapeutic recommendations. Considering that one of the major concerns of physicians is the treatment of chronic diseases (such as asthma, diabetes and heart disease), where the treatment requires ongoing and close medical supervision over the long term, the existence of programs capable of assisting and especially optimizing these treatments becomes essential. Such tools not only increase the number of patients who can be effectively managed but also enhance the overall success rate of medical interventions [20].

According to Santos [18], there are five key sectors within the medical field where the application of AI tools and techniques is expected to drive significant positive changes in health outcomes. These sectors are detailed in Table 1.

The advantages offered by AI in medicine are potentially enormous. These tools can be applied in numerous important scenarios, such as monitoring pregnancy progress, predicting patient turnover in hospital settings, and identifying risks associated with certain drug interactions. Therefore, it can be stated that the benefits for both hospital institutions and medical professionals are substantial, as illustrated in Table 2.

The instruments associated with AI in medicine can play a significant role in evaluating test and exam results to optimize prescribed medication dosages and also in encouraging patients to modify certain behaviors by reminding them of the importance of physical exercise, proper diets, consistent sleeping schedules, and, more broadly, habits that promote a better quality of life. The most anticipated applications in medical studies are directed towards data management, digitized records, and personalized real-time health monitoring, where information is transmitted from wearables and smartphones [18]. Real-time analysis is critically important in cases where urgent results are needed, as delays in professional evaluation can be decisive in either treatment failure or worsening of delicate clinical conditions. It is well known that in many cases, when doctors conduct tests requiring relatively long waiting periods, it becomes challenging to determine the ideal dosage for the patient and to manage their conditions effectively over time.

Unlike traditional treatments, which tend to incur higher costs due to the need for more specialized labor, the emerging AI-driven healthcare model is fundamentally shaped by information drawn from extensive databases. Many new health service models will depend on data analysis platforms integrated with agile AI tools, enabling them to deliver recommendations and support more rapidly.

In addition to the systems used for data measurement and diagnosis, machine learning is an important tool in research development, significantly contributing to the analyses conducted [18]. Consequently, many studies have already been performed focusing on diagnoses aimed at solving specific problems. Data regarding accurate diagnoses are generally accessible through medical records and specialized hospitals, either overall or within specific diagnostic departments, and this information can feed the databases utilized by AI to enhance diagnostic accuracy.

In sum, the clinical integration of these AI systems transcends simple image analysis. In contemporary radiological workflows, AI acts as an ‘intelligent triage’ tool, automatically prioritizing urgent cases, such as intracranial hemorrhages or pneumothorax, in the radiologist’s worklist. Additionally, it automates repetitive tasks like volume segmentation and longitudinal comparison of tumors, allowing the physician to focus on complex diagnostic synthesis. This integration transforms the traditional linear workflow into a collaborative human–AI environment, potentially reducing diagnostic turnaround time and physician burnout [21,22]

Since their inception, machine learning algorithms have been designed for the analysis of medical datasets, providing fundamental elements essential for comprehensive data interpretation. More modern institutions are equipped with monitoring and data collection devices integrated into information system networks. There are specific requirements that every machine learning system must satisfy to be employed in the development of applications related to medical diagnosis and other clinical tasks—as detailed in Table 3 [18].

In relation to deep learning applications in medicine, it is an extremely useful solution for identifying patterns within large biological datasets. Additionally, deep learning technology enables the analysis of medical images such as X-rays, magnetic resonance and tomography to facilitate digital diagnosis of specific pathologies—for example, cancer and diabetic retinopathy. Regarding cancer, its use is particularly relevant in detecting lung and breast cancers, as well as in identifying fractures and tumors through image recognition techniques [20].

The technology of medical imaging, such as computed tomography, magnetic resonance imaging, and X-rays, generates a large volume of data that radiologists must examine and evaluate within a limited time frame. Radiologists are also required to assess factors beyond the images themselves and, in some cases, operate within areas of uncertainty based on unproven hypotheses. In this context, deep learning-based tools can assist diagnosis by utilizing complex pattern recognition methods to identify abnormalities in medical exams and images—such as pulmonary nodules and cerebral aneurysms. These tools can also be applied in preventive medicine; for example, mammography can help detect breast cancer by evaluating irregular structures like microcalcification clusters and hyperdense tissue formations. It is important to emphasize that such tools are designed to support physicians, not to replace them. The final responsibility for image interpretation and diagnosis always remains with the physician [19].

Natural language processing (NLP) can deliver significant benefits when applied to the Health and Welfare sectors. Virtual assistants that utilize NLP technology are increasingly employed in this context. For example, the Lark application has been highlighted for its positive impact on managing chronic diseases. NLP is recognized as an important tool in healthcare, especially when integrated into mobile applications. Moreover, it can facilitate the retrieval and analysis of information from observations, electronic patient records, medical examinations, and patient histories [18].

It is observed that the use of AI tools in accessibility plays a highly significant role. For example, a blind person needs assistance with devices that provide written information. If these devices are equipped with AI capable of understanding natural language, both visually impaired and other disabled individuals can receive assistance independently, communicating directly with the devices throughout the process without requiring a mediator. Over the past decade, there has been widespread growth in studies focused on information technology in health, not only within academia but also across the professional field, highlighting the increasing importance of information technology in medicine and healthcare as a whole. The extensive use of information technology in health has generated and continues to produce a massive flow of patient data. This data, which may originate from medical institutions, health insurers, and laboratories, constitutes an important database that, when analyzed by AI, can substantially contribute to the management and prediction of new cases [23].

The efficacy of AI in evaluating radiological images has been consolidated through robust empirical studies. Landmark research, such as the MASAI trial by Lång et al. [24], demonstrated that AI-supported screening increased cancer detection by 20% while reducing workload by 44.3%. Similarly, Ardila et al. [21] showed that deep learning models can outperform specialists in lung cancer detection, while the CheXpert project and studies by Kuo et al. [25,26] validated AI’s role in emergency triage for chest and brain pathologies. Furthermore, the work of Pickhardt et al. [27] highlighted the potential for opportunistic screening by extracting automated biomarkers from routine CT scans. These large-scale data present highly valuable information for the medical and scientific communities as a whole, supporting decision-making processes and contributing to the reduction in costs and risks for patients. Their analysis offers significant benefits to medicine and its patients, being recognized by specialists in the field as one of the most important technologies applied in health institutions [18].

However, the increasing sophistication of these tools introduces new layers of technical and professional complexity that extend beyond clinical utility. While AI enhances diagnostic efficiency, its integration into radiological reporting challenges the traditional boundaries of medical practice, particularly regarding the inherent opacity of advanced algorithms and the shifting demands on physician expertise. These emerging challenges, ranging from the technical ‘black-box’ to the evolving requirements for professional training, underscore the transition from purely clinical applications to the broader regulatory and bioethical landscape. Consequently, the following section examines the legal frameworks and ethical guidelines necessary to govern the responsible and transparent use of AI in modern medicine.

## 4. Artificial Intelligence in Medicine: Regulation and Bioethical Issues

The advancement in algorithm development, along with greater ease of access to computational resources, now enables AI applications to be integrated into medical decision-making tasks with highly promising results [8,10]. The use of AI techniques in medical imaging is a major area of focus for research teams worldwide. Deep learning algorithms are employed in mammography for diagnosing intestinal cancer, in radiographs for detecting pulmonary nodules, in magnetic resonance imaging for segmenting cerebral tumors, and in supporting the diagnosis of neurological diseases such as Alzheimer’s disease [28,29,30,31,32]. However, some bioethical challenges must be carefully addressed and safeguarded against.

AI systems, including medical and radiological devices, provide sensitive and critical services that, when performed by physicians, require specialized training and formal certification [9,12,33,34,35]. These applications in the medical field raise important bioethical concerns regarding the procedures executed by AI systems, as well as the existing methodologies and techniques to ensure compliance with ethical and professional standards [36]. One primary concern involves the massive datasets required for training these models, which present persistent challenges for data privacy and security, particularly regarding the risk of patient re-identification even in anonymized datasets [37]. Furthermore, the shift toward deep learning-based multi-modal image fusion and multi-level reasoning for automated medical report generation [38,39] makes the decision-making process less transparent to the human operator. This ‘black-box’ nature of AI challenges the principle of explainability, as radiologists must understand the algorithmic logic to fulfill their duty of care. To address this, using frameworks such as Bloom’s Taxonomy and Outcome-Based Education is essential to identify cognitive weaknesses and ensure that practitioners develop the critical competencies necessary to oversee AI outputs ethically and securely [40,41].

Notably, one significant concern is that algorithms may inadvertently replicate human biases in decision-making processes. It is possible that certain ethical biases could be unintentionally embedded within medical algorithms. AI applications introduced in non-medical fields have already demonstrated the potential to make problematic decisions that reflect biases inherent in the data used for their training [42]. Recently, a program designed to assist judges by predicting the risk of repeat offenses exhibited a troubling tendency toward discriminatory outcomes [43]. This example highlights the urgent need for regulatory and policy frameworks governing the use of AI, especially in complex healthcare scenarios where accurate diagnosis and patient management decisions may be controversial and carry significant consequences.

The absence of a clear, stable, and universally accepted definition or conceptualization of AI complicates the development of effective political and regulatory frameworks. Furthermore, there is debate over whether current policies adequately capture the social efforts needed to steer AI development in alignment with the public interest [44]. Thus, it is important to note that not all AI programs used in healthcare are classified as medical devices. Programs that analyze large amounts of data to generate knowledge about a disease or condition, rather than directly guiding treatment decisions for individual patients, may not constitute a medical effect and therefore are not necessarily considered medical devices [45]. This distinction essentially separates research-focused programs, which enhance medical knowledge and promote health advances, from those intended for direct clinical use.

There are two main approaches that authorities can use to regulate AI systems effectively. One is the Precautionary Principle, which imposes limits or even prohibitions on certain applications due to potential risks. This means such applications may not be tested, as authorities seek to prevent possible worst-case scenarios. The other approach is known as Permissionless Innovation, which allows innovations to be explored freely, without prior restrictions, and addresses specific concerns only as they arise [46].

These premises about AI regulation in medical devices highlight, succinctly, the controversial nature of the topic and how it often relies on subjective guidelines and variable interpretations by regulatory authorities. Rapid approval by competent agencies for medical devices incorporating AI provides greater agility for manufacturers in generating revenue and offers physicians quicker access to more effective tools. However, the primary objective of introducing new devices to the market should always be to enhance the prevention, diagnosis, and treatment of diseases, ultimately aiming for a positive impact on patient outcomes.

It is extremely important to develop an international policy framework for the governance of AI in medicine, including in the field of radiology. Therefore, approval systems for new medical devices must create clear pathways for innovative technologies to reach the market, while simultaneously ensuring that patients receive safe, high-quality, and efficient care from both healthcare professionals and medical equipment. To achieve these objectives, European countries and the United States have adopted distinct regulatory approaches [47].

In the European Union, the definition of a medical device is provided by Article 1(2) of Directive 93/42/EEC, which states that the term “medical device” applies to any instrument, apparatus, appliance, software, implant, reagent, material, or other article intended by the manufacturer to be used on human beings for purposes including diagnosis, prevention, monitoring, treatment, or alleviation of disease, among others [48]. This definition is supported by the MedDevs, which are non-legally binding guidelines developed by the European Commission to assist stakeholders in complying with legislation related to medical devices [49].

The current European regulatory regime is established through three directives related to medical devices, which require manufacturers to ensure that their products are suitable for their intended purposes. This means that manufacturers must comply with a set of essential requirements outlined in the European directives. Depending on the risk classification of the device, once these essential requirements are fulfilled, the manufacturer or a notified body may be subject to evaluation; a notified body is an accredited, independent certification organization appointed by competent authorities of European Union member states [48,50,51].

This regulatory framework has been updated through the introduction of the new medical devices regulations (MDRs) and the new regulations concerning the in vitro diagnostic medical devices (IVDRs). Both regulations entered into force on 25 May 2017. The MDR became applicable on 26 May 2022. Unlike the previous directives, the MDR and IVDR are regulations, which means they apply directly and immediately across member states without the need for national governments to enact implementing legislation [52,53,54].

This reform originated from the recognition that the existing directives, established in the 1990s, are no longer adequate to address the challenges posed by evolving new technologies, including AI systems. Some of the key features of this reform include: a broad scope that covers a wide range of products; expanded liability for defective products; strengthened requirements for clinical data; improved traceability of devices; enhanced oversight and monitoring of notified bodies; and greater transparency by making information on medical devices publicly accessible [48,50,51,54,55].

To cite the most recent regulations, the AI Act stands out, adopted by the European Parliament on 13 March 2024, and by the Council of the European Union on 13 June 2024 (with entry into force on 1 August 2024) [56]. This is the first comprehensive regulation on AI in the European Union, which adopts a risk-based approach to classify AI systems (unacceptable, high, limited, and minimal), aiming to promote trustworthy innovation, protect fundamental rights, health, and safety, and establish obligations such as transparency, risk management, and human oversight—especially for general-purpose AI models. Another relevant initiative is the Framework Convention on Artificial Intelligence, Human Rights, Democracy and the Rule of Law, adopted by the Committee of Ministers of the Council of Europe on 17 May 2024, and opened for signature on 5 September 2024 [57]. This international treaty, the first legally binding one on AI and open to global adhesion, seeks to ensure that activities throughout the AI lifecycle respect human rights, democracy, and the rule of law, through principles such as non-discrimination, transparency, accountability, and risk mitigation, overseen by a Conference of the Parties.

In the United States, regulatory approval that would permit machines to perform the tasks of trained radiologists remains a significant and unresolved challenge. The extensive testing and rigorous effort required to obtain clearance from the Food and Drug Administration (FDA) to allow machines to provide critical interpretations of imaging studies without human radiologist involvement can be overwhelming. By the end of 2016, the 21st Century Cures Act clarified the scope of the FDA’s regulatory authority over healthcare software, specifying that a medical device is any instrument, apparatus, implement, machine, or other related article intended for use in the diagnosis of diseases or other conditions, or in the cure, mitigation, treatment, or prevention of disease in humans, or intended to affect the structure or any function of the human body [58,59]. However, the approval of AI systems by regulatory bodies in any country of the world must be preceded by an ethical impact evaluation, due to the overwhelming ethical issues to be considered [60].

To conclude this section, it is worth mentioning that accountability remains a complex legal gray area. In both EU and US jurisdictions, the ‘human-in-the-loop’ doctrine prevails, meaning the radiologist maintains ultimate legal liability for the final report. However, if an error stems from an inherent algorithmic bias that was undetectable by the physician, current tort law struggles to allocate responsibility. While the EU AI Act moves toward stricter product liability for ‘high-risk’ AI developers, the radiological community faces the risk of ‘automation bias’—where excessive trust in the machine leads to clinical oversight—further complicating the determination of negligence in malpractice litigation [61].

## 5. Conclusions

It has been found that expectations around AI are connected to its evident evolution in response to new social and market scenarios. The primary objective of using AI in healthcare to ensure higher-quality medical care was examined, ultimately contributing to the fundamental goal of preserving and enhancing patient life. How information technologies are transforming the fields in which they are applied was determined, with AI emerging as the leading technology in medicine.

It was pointed out that, although the application of AI in medicine and radiology faces numerous challenges, it is essential to recognize that society as a whole increasingly relies on this technological resource—especially considering that healthcare directly involves human lives. Issues such as the creation of new policy frameworks, strengthened data protection regulations, robust cybersecurity measures, the discussion of complex accountability topics, and questions regarding patient trust in AI-based medical systems were analyzed and must be addressed urgently. For this, it is crucial that AI medical resources are developed according to strict bioethical standards, providing clear guidance for healthcare professionals and stakeholders.

It was verified that the design of AI is not intended to replace doctors, but rather to support and enhance their decision-making, since health professionals often cannot make an accurate diagnosis simultaneously while the patient is undergoing treatment. It has been found that the misuse or unethical application of this cutting-edge technology can pose significant risks. Therefore, patients, specialists, and regulatory authorities must collaborate closely to ensure that the use of AI is neither misleading nor unethical.

Thus, one of the most important goals in the application of AI in medicine and radiology is to guarantee that these technological resources are genuinely relevant, clearly describing in detail their potential, usefulness, and value in relation to improving people’s lives.

It was concluded that AI makes a positive contribution when applied to the medical field, as it supports both specialists and non-specialists in decision-making, reducing the incidence of diagnostic errors. It also increases the likelihood of detecting pathologies before they reach critical stages, thereby helping to preserve patient lives and supporting the work of healthcare professionals.

In summary, this study analyzed the main bioethical issues related to radiological reports generated by AI. We emphasize the need to develop guidelines and legislation, as well as best practices for the use of AI in radiology. Furthermore, particular attention must be paid to the progressive implementation of the 2024 and 2025 legal frameworks, as their practical enforcement and judicial interpretation will continue to redefine the ethical landscape of medical AI throughout 2026 and beyond. Finally, it is recommended that additional research be conducted to deepen and strengthen this topic, considering societal evolution, market dynamics, legislation, and the perspectives of leading authors in the field of bioethics.

## Figures and Tables

**Table 1:** Health sectors that could benefit from artificial intelligence.

Sector	Advantages Promoted by the Use of Artificial Intelligence
Care management	Enables the optimization of personalized treatment plans and budget projections, helping to close gaps commonly present in generalized care approaches.
Population management	Facilitates the identification of health risks at the macro level, supporting strategies for public health management and promoting preventive measures and quality of life throughout society.
Self-management of the patient	Enables the development of programs and software that encourage personalized self-care, supporting patients in monitoring and modifying health-related behaviors.
Systems projects	Streamlines processes in both clinical therapies and insurance operations, leveraging data analysis to improve care outcomes and reduce costs.
Decision Support	Assists physicians in making evidence-based decisions (such as adjusting drug dosages based on lab results) and supports radiologists in identifying tumors and other pathologies by providing access to up-to-date research and best practices.

Adapted from Santos [18].

**Table 2:** Advantages promoted by artificial intelligence in medicine.

Advantage	Description
Update in Real Time	Some equipment utilizing artificial intelligence systems can immediately alert doctors and health professionals to any changes in a patient’s clinical condition, enabling faster response.
Data Stored in the Cloud	To reduce costs associated with physical storage, system data is stored in the cloud, promoting better organization, space optimization, and easy access to databases.
Best Diagnosis	Artificial intelligence supports the diagnosis of pathologies by accelerating the diagnostic process, providing safer analyses, and improving the interpretation of radiographs, magnetic resonance and tomography, which often require time to confirm.
Aid in Telemedicine	Telemedicine involves the use of information and communication technologies to obtain information and connect with remotely located professionals. It has proven to be an effective tool, especially in smaller health institutions. With artificial intelligence assistance, for example, mammogram results can be analyzed, and reports issued remotely, enhancing access and efficiency.
Association of Symptoms with Possible Diseases	Sometimes symptoms correspond to multiple possible diagnoses, making final conclusions complex and costly. Artificial intelligence can automatically cross-reference symptoms with a patient’s history within the system, contributing to faster and more precise diagnoses.

Adapted from Santos [18].

**Table 3:** Requirements for a machine learning system to be efficient.

Requirement	Description
Good performance	The algorithm must have the capability to deliver meaningful results by effectively utilizing available data, demonstrating high accuracy in the diagnosis performed.
Performance against insufficient data	In medical diagnosis, patient records are often incomplete—for example, a patient may not know their blood type. The algorithm should be able to handle incomplete information appropriately and treat problems based on the data it receives.
Knowing how to deal with erroneous information	Medical data can contain errors. Learning algorithms need to be robust enough to identify and work around such inaccuracies.
Clarity in the diagnosis	The information presented must be clear and objective to prevent misinterpretation by doctors or patients, ensuring that automatically generated data positively supports the diagnosis. This is crucial as doctors may sometimes overlook or omit critical details in a case.
Justification	The system must provide a well-substantiated explanation of the diagnosis results to build trust among medical professionals. This explanation should be detailed yet concise to maintain objectivity and avoid skepticism.
Decrease in the number of tests for reliable diagnosis	Since patient data collection can often be extensive and time-consuming (potentially delaying treatment), the algorithm should be capable of producing a reliable diagnosis based on a minimal but credible amount of patient information.

Adapted from Santos [18].

## Data Availability

No new data were created or analyzed in this study.

## Abbreviations

The following abbreviations are used in this manuscript:

AI	Artificial intelligence
NLP	Natural language processing
MDRs	Medical devices regulations
IVDRs	In vitro diagnostic medical devices
FDA	Food and Drug Administration
